# Engaging ‘hard to reach’ groups in health promotion: the views of older people and professionals from a qualitative study in England

**DOI:** 10.1186/s12889-019-6911-1

**Published:** 2019-05-23

**Authors:** Ann E. M. Liljas, Kate Walters, Ana Jovicic, Steve Iliffe, Jill Manthorpe, Claire Goodman, Kalpa Kharicha

**Affiliations:** 1 Primary Care and Population Health, University College London, London, UK; 2 Social Care Workforce Research Unit, King’s College London, London, UK; 3 Health and Social Work, University of Hertfordshire, Hatfield, UK

**Keywords:** Ageing, Health promotion, Inequalities, Older people, Oldest old, Ethnicity, Deprivation

## Abstract

**Background:**

Older people living in deprived areas, from black and minority ethnic groups (BME) or aged over 85 years (oldest old) are recognised as ‘hard to reach’. Engaging these groups in health promotion is of particular importance when seeking to target those who may benefit the most and to reduce health inequalities. This study aimed to explore what influences them practicing health promotion and elicit the views of cross-sector professionals with experiences of working with ‘hard to reach’ older people, to help inform best practice on engagement.

**Methods:**

‘Hard to reach’ older people were recruited through primary care by approaching those not attending for preventative healthcare, and via day centres. Nineteen participated in an interview (*n* = 15) or focus group (*n* = 4); including some overlaps: 17 were from a deprived area, 12 from BME groups, and five were oldest old. Cross-sector health promotion professionals across England with experience of health promotion with older people were identified through online searches and snowball sampling. A total of 31 of these 44 professionals completed an online survey including open questions on barriers and facilitators to uptake in these groups. Thematic analysis was used to develop a framework of higher and lower level themes. Interpretations were discussed and agreed within the team.

**Results:**

Older people’s motivation to stay healthy and independent reflected their everyday behaviour including practicing activities to feel or stay well, level of social engagement, and enthusiasm for and belief in health promotion. All of the oldest old reported trying to live healthily, often facilitated by others, yet sometimes being restricted due to poor health. Most older people from BME groups reported a strong wish to remain independent which was often positively influenced by their social network. Older people living in deprived areas reported reluctance to undertake health promotion activities, conveyed apathy and reported little social interaction. Cross-sector health professionals consistently reported similar themes as the older people, reinforcing the views of the older people through examples.

**Conclusions:**

The study shows some shared themes across the three ‘hard-to-reach’ groups but also some distinct differences, suggesting that a carefully outlined strategy should be considered to reach successfully the group targeted.

**Electronic supplementary material:**

The online version of this article (10.1186/s12889-019-6911-1) contains supplementary material, which is available to authorized users.

## Background

Worldwide the population is ageing due to increased life expectancy [1]. Advanced age increases the risk of chronic conditions and disability leading to age-related decline in health and wellbeing [2]. Prevention of age-related chronic diseases and promotion of health to maintain physical and cognitive function in later life can reduce the risks of loss of independence [1]. Health promotion and disease prevention strategies to reach disadvantaged sub-groups of older people are of particular importance to target those who may benefit the most and to reduce health inequalities in later life [3]. ‘Hard to reach’ (also known as seldom heard) groups within the older population are likely to be under-represented in health promotion activities and include the oldest old (aged 85 years and over), older people from black and minority ethnic (BME) groups and older people living in deprived areas [4]. Compared to the general older population, these groups have more health problems and health care needs [5, 6]. These ‘hard to reach’ older people are therefore of particular interest and were the focus of this study. Conducting research that involves the oldest old presents unique challenges as many have reduced cognitive and sensory capacity, affecting communication [6]. However, increasing evidence suggests that the oldest old can benefit more than younger older people from health interventions [7, 8], making the oldest old an important target group for health promotion. Targeting this group is also important as they form the fastest growing segment of the population [9]. Similarly, the number of BME older people in Western societies is rapidly increasing [10], though this is seldom reflected in research studies of older people [11, 12]. Previously reported unique challenges essential for engaging BME older people in health promotion include addressing language barriers and having a culturally sensitive approach [13]. Further, older people who live in deprived areas are more likely to experience multi-comorbidity and this tends to occur 10–15 years earlier compared to older people who live in affluent areas [14]. Earlier research has reported that costs, inadequate access and lack of public transport can negatively affect participation in health promotion among older people from deprived areas [15].

Our recent systematic review on engaging ‘hard to reach’ older people identified numerous facilitators and barriers for recruiting and engaging the oldest old, older people from BME groups and older people living in deprived areas to research on health promotion [16]. The review revealed that some facilitators and barriers were shared across all three sub-groups but there were also differences between the groups, such as location for recruitment and preference of individual versus group sessions. However the systematic review showed that the majority of previous studies were undertaken in the United States (US), and previous studies have primarily focused on physical activity in older people from BME groups [17–19], while other health promotion topics and ‘hard to reach’ sub-groups, including those living in deprived areas and the oldest old, have not been extensively investigated. To our knowledge, no previous study has explored the views of professionals from multiple sectors with experience of working with these ‘hard to reach’ older people in health promotion. A more comprehensive understanding of the views and attitudes of older people ‘hard to reach’ and the views of professionals with experience working with them may help to reduce inequalities in uptake of healthy ageing activities. Well-targeted health promotion strategies may help ensure resources for improving the health of older people are used effectively and efficiently. Therefore, the aims of the current study are: 1) to explore what influences ‘hard to reach’ older people practicing health promotion in later life; and 2) elicit the views of cross-sector professionals with experience of working with these sub-groups of older people, to help inform best practice on engagement of ‘hard to reach’ older people in health promotion.

## Methods

### Study population, sampling and data collection

Study participants consisted of: 1) community-dwelling older people from three ‘hard to reach’ groups including those aged ≥85 years, older people from BME groups, older people living in deprived areas, and; 2) of professionals with experience working with one or more of these sub-groups of older people. Recruitment and data collection took place between December 2014 and August 2015.

#### Sampling and data collection of ‘hard to reach’ older people

‘Hard to reach’ older people were recruited from two primary care practices, one BME community group and one day centre for older people. All recruitment sites were located in north London. Our recruitment strategy evolved throughout the study to identify participants until data saturation was reached. In the first round of recruitment, the primary care practices were asked to identify patients aged ≥85 years and patients aged ≥65 years from BME group and/or living in deprived area (based on the postcode of the patient’s home address) who did not attend annual flu vaccination or blood pressure check in the last two years from electronic searches of records excluding patients on the palliative care register. Participants were also excluded if they lived in care homes, were terminally ill or were on the quality outcomes framework for documented dementia. The participating practices’ views were sought on how best to contact eligible patients. Eligible participants were approached by the practices via post or face-to-face opportunistic recruitment. The participant information sheet was translated into different languages and in larger font size that was provided on request. Interpreters were arranged for interviews / focus group with participants who did not have English as their first language or their spoken English was not sufficient enough to participate. Using interpreters enables participants who do not speak English to be included in the study, potentially providing additional perspectives. However, if the translation is poor, the meanings of the participant’s words may be lost, which may generate misleading conclusions of the results [20]. Such risks seemed, however, fairly small in this study as the findings from the participants who required an interpreter mainly confirmed what other participants already had reported. Additional specific needs of participants, including sensory impairments and mobility needs, were considered too. Potential participants were encouraged to discuss the research with the organisational leads, friends, family or the research team including for any clarification about what their involvement in the research would mean. Telephone reminders were made to non-responders. Twelve participants were interviewed from the first round of recruitment in primary care.

Purposive sampling was used in a second round of recruitment to target the oldest old and older people from BME groups. To increase diversity with respect to these groups, recruitment was carried out through a day centre for older people and a BME community group. For this second round of recruitment, information on having attended flu vaccination or blood pressure check in the last two years was not considered. Three participants aged ≥85 years were recruited from the day centre and an additional four participants were recruited from the BME community group.

Two female researchers with experience of interviewing older adults carried out the interviews. They had backgrounds in psychology and public health, one was from a BME group. They introduced themselves as researchers from the University, with no specific information on their background. The date, time and venue of each interview were decided based on the participant’s availability and preference. Participants recruited from primary care were interviewed in their homes, those from the day centre were interviewed at the day centre in a quiet room. One man aged ≥85 years wanted his wife to sit next to him during the interview at their home as he thought his memory had deteriorated and prompted his wife to expand on a few of his responses; other interviews were conducted one-to-one.

The manager of the BME community group suggested that members were more likely to participate in a group discussion at the day centre than a one-to-one interview in their homes. The organisation invited members and arranged for an interpreter to help take consent and translate the discussion from English to the main language spoken by the participants. At the focus group, the researcher asked one question at a time, in English, directing questions to the participants rather than the interpreter. The interpreter translated the question and then translated the responses given by the participants back into English. The semi-structured interviews and focus group lasted for between 45 and 90 min and were audio-recorded and transcribed verbatim with consent. Field notes were written after each data collection.

A topic guide (Additional file 1) was developed to address the research question. Topics were inspired by and derived from previous research and discussed within the research team. The research team included members with expertise in ageing, general practice, nursing, social care, psychology, public health, and lay members. The questions in the interview guide were followed-up with prompts as necessary to elicit a deeper exploration of participant experiences and views. Topics included the participants’ views and experience of health promotion, what helps or hinders them staying well, support needed to stay healthy in later life, and decision-making around seeking help for health. Demographic data on age, gender, ethnicity, living arrangements, housing status, level of education and income (pension, benefits) were collected.

Data analysis was commenced alongside data collection. Data collection was continued until saturation on the main themes was reached and no new views of challenges to the interpretation of the themes were identified. This occurred after the second round of recruitment when a total of 19 participants had been interviewed (*n* = 15) or participated in the focus group (*n* = 4).

#### Sampling and data collection of professionals

Through online searches for people from various sectors working with any of the ‘hard to reach’ groups and snowball sampling, starting with individuals identified via the research team’s networks, we identified 157 professionals with valid email addresses who had relevant experiences. The professionals were based across England and from various sectors including academic, voluntary, health, local authority and social care. Identified professionals were emailed information about the study and asked to reply if interested in participating. A reminder was sent two weeks later to those who had not replied. Professionals who replied to the email were provided with a web-link to a structured questionnaire with a series of open questions asking them to report on facilitators and barriers for each of the ‘hard to reach’ groups with whom they had experience of working (Additional file 2). Responses were entered as free text. The web-link was available for 30 days and data were downloaded at the end of the time period. A total of 44 professionals replied to the email and were sent the web-link to the online questionnaire which was completed by 31 professionals. No reminder emails with the web-link were sent to those who agreed to participate as the survey was anonymous with no opportunities to identify and separate those who had and those who had not completed the survey. Participants provided consent prior to survey completion. Geographical location of those who replied was recorded, however, further data on geographical location was not collected part of the survey.

### Data analysis

A Thematic Framework analysis was used to analyse the data [21]. All the transcripts from the interviews and focus group with older people were read independently by three researchers and all members of the research team read at least two transcripts each. Patterns and themes emerging from the data were then discussed as a group, refined further and organised into a thematic framework. A matrix was developed from the framework into which all data were systematically apportioned using Microsoft Excel 2013 software (v15.0) [22]. The majority of older people belonged to more than one of the three groups. Data were analysed both within each of the groups and across groups to identify both common and group specific themes; this process was facilitated by the use of the framework analysis matrix.

Free-text responses to the professionals’ survey were initially analysed separately to the data from the older people. Data were read by three researchers who independently identified and collectively agreed themes. To establish if the findings resonated with the experience and knowledge of the research team and their transferability to other settings, themes were discussed with the entire research team including lay members. Themes from the professionals’ data were considered alongside those from the older people. Interpretations were drawn collectively within the team.

## Results

### Characteristics of participants

Table 1 shows the characteristics of the 19 ‘hard to reach’ older people aged ≥65 years (8 men, 11 women) who participated in the study. In total, 5 participants were aged ≥85 years, 12 were from a BME group (4 Black African Caribbean, 4 Bangladeshi, 4 White Europeans (including 3 Irish and 1 Spanish)) and 17 participants were from deprived areas. The majority of participants belonged to more than one of the groups of interest (see Table 1 for details).

**Table 1:** Characteristics of participating older people

ID	Data	‘Hard to reach’ group	Gender	Age in years
Int1	Interview	Oldest old	Male	≥85
Int2	Interview	Oldest old, deprived area	Female	≥85
Int3	Interview	Oldest old, BME, deprived area	Female	≥85
Int4	Interview	Oldest old, BME, deprived area	Female	≥85
Int5	Interview	Oldest old, BME, deprived area	Female	≥85
Int6	Interview	BME	Female	65–84
Foc1	Focus group	BME, deprived area	Female	65–84
Foc1	Focus group	BME, deprived area	Female	65–84
Foc1	Focus group	BME, deprived area	Female	65–84
Foc1	Focus group	BME, deprived area	Female	65–84
Int7	Interview	BME, deprived area	Male	65–84
Int8	Interview	BME, deprived area	Male	65–84
Int9	Interview	BME, deprived area	Male	65–84
Int10	Interview	BME, deprived area	Female	65–84
Int11	Interview	Deprived area	Male	65–84
Int12	Interview	Deprived area	Male	65–84
Int13	Interview	Deprived area	Male	65–84
Int14	Interview	Deprived area	Male	65–84
Int15	Interview	Deprived area	Female	65–84

Of 44 professionals who received the web-link to the survey, 23 were based in London, 7 in the Midlands and East England, 10 in North England and four in South England. Table 2 presents the characteristics of the 31 out of the 44 professionals who responded to the survey, working in the academic sector (*n* = 11 including researchers/lecturers and directors), local authority (*n* = 9 including practitioners, commissioners, managers and city councillor), voluntary sector (*n* = 7 including project officers, managers and trustee), the National Health Service (NHS) (*n* = 2 practitioners) social care (n = 1 manager) and one professional reporting work-experience from multiple sectors. Some professionals had worked with and provided information about more than one ‘hard to reach’ group resulting in 18 professionals responding to the questions about their experience from working with the oldest old, 16 reporting about their experience from working with older people in deprived areas, 16 reporting on their experience from older people from BME groups and nine sharing their experience of working with other ‘hard to reach’ groups including older people with certain disabilities, comorbidities, poor health literacy, those who were home-bound and those who were socially isolated.

**Table 2:** Characteristics of participating professionals

ID	Job sector	Job role	‘Hard to reach’ group of experience
Pro1–11	Academic	9 researcher/lecturer, 2 director	7 oldest old, 6 BME, 4 deprived area
Pro12–20	Local authority	2 practitioner, 5 manager/director, 1 commissioner, 1 city councillor	2 oldest old, 6 BME, 7 deprived area
Pro21	Multiple sectors	1 ‘health promoter’	Deprived area
Pro22–23	NHS	2 practitioner	2 oldest old, 1 BME, 2 deprived area
Pro24	Social care	1 manager	Oldest old
Pro25–31	Voluntary/third sector	3 project officer/coordinator, 3 manager/director, 1 trustee	6 oldest old, 3 BME, 5 deprived area

Below the findings of older people’s views and the views of the professionals are presented together emphasising commonalities and differences where possible. The unique and overlapping themes of the three ‘hard to reach’ groups are outlined in Table 3. The findings should be interpreted with caution as similarities and differences reported have been identified through comparison between the three ‘hard to reach’ groups by the research team.

**Table 3:** An overview of the overlapping and unique themes of the three ‘hard to reach’ groups

Themes	Oldest old	BME	Deprived area
Context of older people’s lives and health	x	x	x
Practicing activities to feel or stay well	x	x	x
The role of others on health and wellbeing	x	x	x
Practicing health promotion requires making an effort	x		
Behaviours to reach advanced age	x		
Withdrawal from health promotion activities	x		
Wish to remain independent		x	
Taking health advice		x	
Language barriers		x	
Scepticism about engaging in health promotion			x
Apathy			x
Opportunities for health promotion in the local area			x

### Themes across all ‘hard to reach’ groups

Themes identified across all three ‘hard to reach’ groups included context of their lives and health, practicing activities to feel or stay healthy, and the role of other people on health and wellbeing.

#### Context of older people’s lives and health

Participants from all three ‘hard to reach’ groups reported on their everyday life including health challenges ranging from problems that they could cope with using medication, such as hypertension, to multiple chronic conditions affecting their everyday life:

*“Not being able to do what you want to do plays a great part in my life, it more or less makes me handicapped, like a handicapped person because I can’t do this, I can’t do that, I can’t … you know? It’s as if my life is not my own now. … . I can’t do anything hardly. I can’t hoover, I can’t make up my bed. Sometimes even to get my clothes on, I can’t do that. I can’t go in the bath on my own. So it’s hard and I cannot rush to do anything.”* (Int10, female, BME, deprived area)

Professionals reported similar views of the health and everyday life for ‘hard to reach’ older people:

*“General lack of physical ability gets in the way. There are things like loss of or difficulty in seeing, hearing. Some may have dementia.”* (Pro27, voluntary/third sector, trustee)

#### Practicing activities to feel or stay well

Most participants across the ‘hard to reach’ groups described their considerations and/or practice of activities that helped them feel or stay well either on their own or with others. Such activities referred to their physical health including being active, using aids such as walking stick, moderating alcohol intake, taking medication and vitamin D supplements, flu vaccination and blood pressure checks. Activities to feel or stay well also included cognitive health including reading papers and doing puzzles, crosswords and Sudoku, and social health and wellbeing including attending church and charity events:

*“Well, in my young days, I didn’t exercise because I was working, but now I come down here for exercise classes, you know, they’re held in the morning.”* (Int5, female, oldest old, BME, deprived area)

*“I mean, you are aware of the benefits of it, of course you are, because otherwise you wouldn’t go, you wouldn’t even think about it. So you know the benefit is ongoing but actually going itself, getting up and going, that’s the main thing.”* (Int1, male, oldest old)

*“I read quite a lot and I struggle with the crossword and do the Sudoku and that sort of thing.”* (Int11, male, deprived area)

#### The role of others on health and wellbeing

Older people across the three ‘hard to reach’ groups reported that other people influenced their engagement in health promotion activities. Among the oldest old, practicing health promotion activities was often facilitated by others including family members, their peers, General Practitioner (GP) and services:

*“Yeah, they [peers] have cars and they come and take me to church.”* (Int3, female)

*“They [patient transport services] take me where I have to go and pick me up and take me home as well.”* (Int4, female)

*“The other thing is that I can’t go out now on my own, unless I’m with my daughter with a stick and I hold her arm.”* (Int2, female)

In addition to family members, many older people from BME groups also talked about peers influencing health promotion actions. For most BME participants, family and peers had a positive influence on health promotion:

*“I’ve spent a lot of time with my granddaughter, she’s eight now. And so she gives you a whole new outlook on life and she does gymnastics, so she has me down on the floor doing some of them with her!”* (Int6, female)

*“A friend introduced me to [the community centre], they were having something and she took me. And then I became a member.”* (Int3, female)

Some reported, however, that family and peers do not always have a positive influence on their older members’ health and wellbeing, including peers dropping out of health promotion classes and family members needing to be cared for:

*“But they [peers from the same BME group and age] just stopped, they just stopped coming [to health class]; they didn’t say why they’re not coming and then in the end, there was just me alone.”* (Int10, female)

*“Now her husband is not well and that’s why she looks after her husband.”* (Foc1, female x4)

To some BME older people it was important for their wellbeing to have someone to talk to and this tended to be a relative or a friend in their community. Several had a number of people whom they chatted with regularly:

*“Sometimes it’s good to have somebody to talk to you, better than taking this rubbish medication.”* (Int10, female)

*“The community church and you go there and meet a friend from your country, you talk and it’s good.”* (Int9, male)

Among older people living in deprived areas, some reported staying in touch with friends mainly by telephone. A few also reported attending the luncheon club for opportunities to socialise.

*“Well, you meet other people. You meet a lot of people who don’t just live in the block (of flats) in the area.”* (Int11, male)

However, others living in deprived areas reported that they themselves and/or others did not want to socialise:

*“I used to go to [adjacent area] to that day centre and they have the little minibus taking you there. I’ve tried to get a few of them involved to go, because they’re not doing anything. But they didn’t want to; I was the only one that the bus picked up from here.”* (Int10, female)

*“I don’t know a good many people. I don’t need to socialise a great deal … . If not at 40 [years of age] I wasn’t particularly worried about making friends with them, why should I do it 20 years later.”* (Int14, male)

One participant also reported negative social encounters due to tensions between older residents of the housing estate: *“There’s one fellow who thinks he owns the building, you know?”* (Int13, male).

The same participant also reported some peers having negative attitudes towards each other: *“Well, you know what people are like: ‘I’m not sitting with her!’”*

Professionals’ views corroborated those of the oldest old. From their experience, engagement in health promotion is often facilitated by peers and providing transport to the venue where the intervention is hold s essential. Some reported that for the oldest old it is important to offer health interventions in their homes:

*“Arrange transport for elders so that they can be picked up from their home”* (Pro4, academic, researcher/lecturer)

*“[To facilitate participation] engaging with this group in their own homes”* (Pro31, voluntary/third sector, director)

Whilst some professionals reported family members and carers supported their older relatives in taking part in health promotion, others had experience of family members not being in favour of such engagement:

*“Attitudes of others. Can be linked to ageism but here expressly meant as concerns of family and careers in older relatives participating”* (Pro3, academic, researcher/lecturer)

Some professionals with experience of working with older people from BME groups reported that the family commonly looks after their older relatives. This could, however, be a burden for the family carer unless they are aware of the support and facilities available:

*“A reluctance to engage with services, families preferring to take care 'of their own' - of course this is encouraged, but along with certain support mechanisms when appropriate would increase carer support and reduce risk e.g. by providing moving and handling equipment rather than a carer lifting a cared-for-person manually.”* (Pro18, local authority, practitioner)

BME older people who thought it was important to have someone to talk to also lived in deprived areas suggesting this finding may also apply to other older people in deprived areas. Professionals with experience of working with older people from BME groups emphasised that addressing social aspects to successfully engage older people in health promotion particularly related to BME older people:

*“The socialising and community aspects of older BME groups is important. For some communities I worked with, particularly Viet Laos Cambodian and African / African Caribbean it would be seen as quite rude to try to 'engage' without FIRST enjoying a meal together.”* (Pro15, local authority, commissioner)

Social isolation among older people living in deprived areas was reported by some professionals too:

*“[I] often come across residents who have no social contact with their neighbours, family or friends. Ill-health often lead to social isolation when close family decease. In some cases residents do not get out and sometimes becomes social isolated. The groups who are not involved with e.g. churches, clubs or other organisation, it can be very difficult to engage with these groups.”* (Pro17, local authority, city councillor)

### Oldest old

Three higher level themes emerged for the oldest old with sub-themes (bold) as follows: practicing health promotion requires making an effort, behaviours to reach advanced age (behaviours and attitudes in earlier life, reacting to health problems, selective actions), and withdrawal from health promotion activities (poor health).

### Practicing health promotion requires making an effort

Some of the oldest old participants described how practicing health promotion required making an effort:

*“I just have to, you know, take my time, like going up the stairs and so on, I have rails to hold onto to go up. I take my time, one at a time, you know?”* (Int3, female)

*“But if I go out with a pusher (mobility aid), I can go out with that on my own, but I can’t put weight on it to go downstairs.”* (Int2, female)

#### Behaviours to reach advanced age

Many examples were given of the oldest old reporting **behaviours and attitudes in earlier life** having an impact in advanced age. Several of them thought that lifelong healthy eating habits and having been busy bringing up a family might have helped them staying healthy. However others thought that living a long life is to some extent due to chance:

*“First of all it means being lucky for a start, not everybody is healthy. It’s something like a lottery, isn’t it?* … *Well, looking back, in hindsight looking back, all the things I could have done to improve my health but I didn’t do it. One was smoking, I smoked all my life, so that was a ‘no-no’, but there again, I grew up in a culture that encouraged you to smoke.”* (Int1, male)

Some of the oldest old were sensory impaired and reported having **reacted to health problems** by undergoing cataract surgery, getting new glasses and hearing aids to address health problems that restricted them in their everyday life. Actions to prevent further falls and maintaining independence included walking and mobility aids to move around the house including when going to the toilet at night or having a bath on their own. The health promotion actions taken were, however, **selective**, mainly based on what they thought needed to be prioritised to function on a day-to-day basis (e.g. visual problems). For example, some reported they knew the benefits of having a regular dental check but had not seen their dentist for years.

#### Withdrawal from health promotion activities

Some of the oldest old reported having stopped doing health promotion activities such as visiting a community centre, attending local events and going out when dark. The most common reason for withdrawing from activities was feeling weaker compared to when they were younger, and feeling vulnerable. Most participants furthermore reported consequences of **poor health** including chronic conditions and mobility problems as obstacles for practicing health promotion outside the home:

*“No, I don’t go out any more when it’s late like that [7 pm], unless my boys are here and they have a show or something and they take me out to it. But I wouldn’t like going out in the evening, late in the evening now.”* (Int3, female)

One participant reported hesitating going to a local charity event without their partner who was too ill to attend:

*“Well, I felt they couldn’t give me nothing at the moment. It was a place … it was, er, (location) and I found it hard to get there and also I didn’t really get to … because I used to go there with my partner, going there on my arm, I didn’t really know anybody, know what I mean? If I go with my partner, I was OK, but going on my own was a different matter altogether.”* (Int1, male)

According to the professionals, to engage the oldest old in health promotion it is essential to visit an array of places where the oldest old are, including their homes and local clubs and groups. Professionals reported poor health being one of the most common obstacles for engagement in health promotion in adults aged ≥85 years. To overcome this, professionals shared their positive experience of providing a telephone service to reach those who did not visit the community centre. It was furthermore suggested that peers, carers and possibly family members should be involved when engaging with the oldest old addressing problems of poor health as someone’s health status relates to both physical, mental and social health:

*“Engagement with their wider social networks, which might include kinship but also community groups. Direct involvement of formal care workers and informal carers.”* (Pro6, academic, researcher/lecturer)

### Older people from BME groups

Four key themes emerged for older people from BME groups: wish to remain independent (motivation to live, support from family to stay independent),, taking health advice, and language barriers.

#### Wish to remain independent

Most participants from BME groups expressed a strong wish to stay as healthy and independent as possible. For some, their **motivation to live** was a reaction to the deaths of their siblings and others:

*“I suppose being independent; that’s the biggest thing to me, losing my independence.”* (Int6, female)

“W*ell why should I go before the younger ones!?”* (Int3, female)

Most participants from BME groups also reported receiving some **support from family to stay independent** including assistance with domestic tasks and transport to, for example, church:

*“My family help with cooking, washing, everything the family can do, you know?”* (Int9, male)

*“Daughter-in-law stays at home, she’s not work, in that way she supports.”* (Foc1, female x4)

#### Taking health advice

Some older people from BME groups who also lived in deprived areas reported relying on healthcare professionals, particularly their GP, for health advice as they trusted them:

*“I don’t take advice from anyone, only my doctor. He knows about all the problems that we have. I wouldn’t trust anyone else; I’d trust my GP because they’re trained for that, they’re qualified in that. So I’ll take their advice any time.”* (Int8, male)

Taking health advice also included attending health check-ups at the GP practice:

*“You get the letter, you go there.”* (Int9, male)

Some participants reported getting health advice from their community centre and from their children. In contrast, some older people from BME groups living in deprived areas were indifferent to health advice from anyone as they believed little could be done about their health due to advanced age:

*“I don’t know, it’s just old age, I don’t know. It’s long term problems.”* (Int10, female)

*“I don’t expect much when I go to the doctor; I know that they cannot do miracles. If you’re old, you are old.”* (Int7, male)

Some participants reported accepting health advice from health professionals for flu vaccination and blood pressure checks but not for their diet as the advice given was not considered culturally appropriate:

*“They [health professionals] tell us boiled vegetables and things, but we’re not eating them. What doctor advise us to boil food, that will not be tasty, that’s the reason not listen to doctor!”* (Foc1, female x4)

Most professionals reported that creating a relationship of trust was vital when engaging with older people from BME groups because of potential barriers working with this group, such as language (presented below) and cultural differences. Some professionals reported experience of overcoming mistrust from BME participants by spending time talking and listening to them:

*“I found it can take time because you need to build trust and understanding of culturally appropriate ways of working.”* (Pro15, local authority, commissioner)

#### Language barriers

Language barriers were reported by some of the older people from BME groups as an obstacle for engagement in health promotion. The focus group participants of whom none spoke English reported that due to their poor English they limited their time outside their home and were more likely to stay indoors. Some participants also reported that not being able to speak the language had caused problems getting the medical help/treatment they needed:

*“Like sometimes when the doctor is speaking, you don’t understand and it’s very important to have some interpreter.”* (Int9, male)

All professionals who had experience of working with older people from BME groups mentioned language barriers as particularly important to address:

*“It is vital to have someone who can speak the languages, or very good translators.”* (Pro2, academic, researcher/lecturer)

### Older people living in deprived areas

Among older people living in deprived areas, four high level themes and five low level themes emerged: scepticism about engaging in health promotion (resistant to health advice, unwilling to use health services and interventions), apathy, and opportunities for health promotion in the local area (free services and transportation, encouragement and continuity, poor literacy).

#### Scepticism about engaging in health promotion

Whilst a few participants reported trust in doctors and other healthcare professionals, others were **resistant to health advice**:

*“I hate people telling me what to do, you know? A bit of advice, fine, but don’t try and force it down me, you know?”* (Int13, male)

Some participants reported their peers or themselves being **unwilling to take up health services interventions**:

*“They [peers of the same age] don’t like to go to the doctors, they don’t like doctors, let alone going to see them! And they hate hospitals. I think because their mothers and fathers and things from many years ago died in hospital, you know, because they didn’t have the facilities. … ‘If I go in there, I shall die; I won’t come out!’ That’s their favourite saying.”* (Int13, male)

*“Well, I’m reluctant to go [visiting GP], but I find I’ve had to accept it over this last year or so, that I have to go when it’s necessary. I don’t … I didn’t think that, er, it was necessary and, er, it’s not part of my life, it hasn’t been part of my life to go to the doctors’ surgery, up until now. So, you know, it was just something that was happening to other people that they have to go, maybe, and I didn’t.” (*Int14, male)

Some participants thought little could be done to improve their health including two participants reporting not telling their GP about their memory problems as they considered it to be part of ageing:

*“I forget things; one hour or two, I put something here, and one hour or two, what did I put? The memory, in the last three years, the memory is not so good, and that’s all. So I manage with what I’ve got left (slight laugh) and it’s nothing the doctor can do, I know, with memory.” (*Int7, male)

When asked if he (Int7) would like to attend a group session to help improve his memory, he replied: *“I’d be interested in, yeah, but I don’t believe that it can be done.”*

To describe the attitudes of older people in deprived areas to engagement in health promotion, professionals used words such as ‘suspicion’. Lack of trust was also repeatedly reported by professionals with work experience of this ‘hard to reach’ group:

*“Lack of trust of those who should be supporting.”* (Pro21, multiple sectors, ‘health promoter')

*“Reluctance to change.”* (Pro18, local authority, practitioner)

#### Apathy

Many older people living in deprived areas reported peers and sometimes themselves having stopped showing an interest in doing things and staying at home doing nothing:

*“I think it’s all the attitude of the mind with them … a lot of them [peers of the same age], I don’t know, they think they’re going to die tomorrow and they’ve got to really closet their self and don’t do anything, you know? Because a lot of them are younger than me”* (Int13, male)

*“A lot of people [peers of the same age] just let themselves go; they just can’t be bothered. It’s the way you think, right, you know, that I think that keeps you young. I know friends of mine, they just sit indoors and they puff and they blow when they go out. I find like a lot of people that I ask to an event and they say, ‘Oh, I don’t want to go to that!’”* (Int15, female)

*“Well, I think there seems to be a mind-set, I would think, of the older people. People when they reach about 50 or 55, or maybe even earlier, they think ‘I’m physically done now, it’s all downhill!’”* (Int14, male)

*“An awful lot of people here, I fear, sit at home all day in their flat. … So I think you’ve got to encourage those who are using these facilities to get other people to draw them in.”* (Int11, male)

Some professionals reported that older people living in deprived areas often experienced a loss of control over their own lives:

*“Apathy, not empowered to think they can make a difference - lack of genuine engagement i.e. coming out to where they are.”* (Pro22, NHS, practitioner)

*“This group often say 'it's too late to change'.”* (Pro18, local authority, practitioner)

One professional reported using other older people who live in deprived areas to ask them about any locally based peers whom they are worried about:

*“I found they often are aware of someone who doesn’t come out much or engage.”* (Pro15, local authority, commissioner)

#### Opportunities for health promotion in the local area

Older people living in deprived areas reported that health promotion was facilitated by **free services and transport**:

*“Well, the services, like I go to the swimming pool and it’s free for me, for over 65s it’s free swimming, which is a very good service.”* (Int12, male)

*“An acquaintance from the keep fit (class), and her husband is coming up to 90 [years of age]. These are very fit people and they get the local bus from the council to take them shopping once a week; so I mean more amenities like that for the elderly that help people to live independently.”* (Int15, female)

Providing free access and transport to facilities was also reported by professionals as enablers for participation in health promotion interventions:

*“Putting on relevant events that are close to where they live. Preferably free and providing a lunch or other refreshments. Getting transport organised for those less able to use public transport.”* (Pro27, voluntary/third sector, trustee)

Some participants reported **encouragement and continuity** of activities would facilitate engagement in health promotion:

*“I mean, my exercise, I’ve got a lot of machines here to do my exercise; if I get some encouragement to start doing something, like go to the gym or something”* (Int8, male)

*“I’d go there one day and we’d be up there, and then next week we’d be over there, and the next week up there, and then they wouldn’t turn up that week. I just gave it up, you know, because I can’t be bothered with things like that.”* (Int13, male)

Among a few older people in deprived areas, **poor literacy** was reported as an obstacle for independent living.

*“It wasn’t language, because he [older neighbour referred to as ‘an English guy’] couldn’t read and write … He knew he’d got a letter, so he would phone me up and say, ‘Could you come and read this letter for me?’ so I used to go over and read it for him. The thing is, with people who can’t read and write, they need advice, because he really needed someone to advise him on how he could get help and nobody visited him. ”* (Int8, male)

Some professionals working with older people living in deprived areas reported poor literacy had as a consequently reduced the amount of written information provided and obtained.

*“[Enable participation by] not too much written info (information) required to fill in before enrolment.”* (Pro23, NHS practitioner)

## Discussion

### Summary

Most older people practiced some health promotion activities part of their everyday life in order to feel or stay well. The oldest old had mixed perceptions on why they had reached advanced age yet all of them practiced health promotion to help them stay well, often facilitated by others and sometimes restricted due to poor health. Most older people from BME groups reported practicing health promotion and had a strong wish to remain independent which was often positively influenced by their social network. Older people living in deprived areas often reported that they themselves or their neighbours of the same age were reluctant to carry out health promotion, conveyed apathy and described little social interaction. The views across professionals from academia, management and practice were consistent with the themes emerging from ‘hard to reach’ older people themselves and both reinforced the views of the older people and provided some additional aspects, generating a greater understanding of ‘hard to reach’ older people’s views on engagement in health promotion.

### What the findings suggest

#### Across the ‘hard to reach’ groups

Older people interviewed across the three ‘hard to reach’ groups reported engaging in health promotion to some extent. This is comparable to other research in the general older population that has reported engagement in various health behaviours to stay well [23, 24]. Participants who were members of the two ‘hard to reach’ groups ‘BME’ and ‘living in deprived areas’ reported conflicting messages on taking health advice from health professionals; some of them only trusted their GP for health advice, some were selective in what advice from health professionals they practised, and some were indifferent to health advice, thinking that nothing could be done due to their old age. Resistance to health advice was also reported by some white older people living in deprived areas suggesting that older people from BME groups and older people living in deprived areas may to some extent overlap due to shared exposure to social and economic disadvantages [25]. Belonging to more than one ‘hard to reach’ group may furthermore contribute to multiple disadvantages that are likely to be complex and interrelated [26]. The comparisons of the findings between the three ‘hard to reach’ groups should be interpreted with caution as they were made by the research team during data interpretation and were not specifically probed during the interviews.

#### Oldest old

All oldest old participants reported practising health promotion (taking exercise, doing puzzles) as well as reacting to health problems by obtaining, for example, hearing aids. Their engagement in health promotion supports previous studies emphasising health benefits among the oldest old from participation in physical, social and intellectual activity [27, 28]. It should be noted however that the response rate to invitation to interview from primary care was low in this group and three of the five people aged 85 years and over were recruited from a day centre. These views therefore are likely to represent a more engaged sub-set of people over 85 years. Our findings further showed that, according to professionals with experience of working with the oldest old, needs and opportunities for health promotion among this ‘hard to reach’ older people should be tailored to the individual to maximise independence and quality of life. Current NHS England policy emphasises a person-centred approach [29], which has potential for early detection of health decline and adjustments to be made. However, it is also known that to date such approach has not been fully implemented [30]. Nevertheless, health promotion interventions designed around individual needs may reduce the risk of people withdrawing from health promotion activities due to poor health, a problem reported in the current study. Such individual needs could potentially be targeted by offering home visits, reported as an important enabler for health promotion of the oldest old in the current study and in a recent systematic review [16].

#### Older people from BME groups

Among older people interviewed from BME groups, their social network played an important role in their lives and professionals with experience working with them reported social aspects being essential to successfully engage them in health promotion interventions. The views of older people from BME groups did not differ between the BME sub-groups. The findings from the survey with professionals working with this group suggested that strong family connections can however result in underuse of care and aids available to facilitate for the family to provide care for their older relative. Older people from BME groups can suffer discrimination in accessing services and may be particularly disadvantaged as the range of services available to BME older people vary considerably across England [31]. Involving family members has previously been reported as particularly important for the oldest old [16], but may be important when engaging older people from BME groups too.

Poor English was reported as a problem to engage in health promotion by participating older people from BME groups and by professionals with work experience of this group. Addressing language difficulties has been reported as a priority in both national and local policies to facilitate use of services and participation in health promotion for older people from BME groups [32]. It is possible that reducing language barriers may furthermore provide opportunities for a more person-centred approach and shared decision-making. Targeting language barriers may play a particularly important role for older people from BME groups as our findings show that they predominately seek health advice from healthcare professionals. The National Service Framework for Older People earlier suggested that healthcare professionals working with older people from BME groups should be aware of, for example, chronic conditions particularly common in certain BME groups to be able to provide relevant health advice and health interventions to older people from different BME groups [31].

#### Older people living in deprived areas

In the current study, some older people living in deprived areas reported themselves or their peers of the same age being reluctant to accept health advice and unwilling to participate in health promotion accompanied by little social interaction. This supports previous studies investigating older people’s attitudes towards health promotion concluding that older people in deprived areas are excluded from involvement in social relationships and activities within their wider communities [23, 33]. Targeting and support older people in deprived areas are therefore essential to improve their health and reduce health inequalities [33]. Behavioural education on goal setting and planning daily habits have the potential to empower vulnerable older people and improve their quality of life [23]. Social support can furthermore positively influence on the process of becoming empowered [34], and contribute to independence and improved quality of life [1]. Nevertheless, older people in deprived areas participating in the current study did not only report lack of social contacts but also exposure to negative social communication, possibly reducing the chances of people attending activities. Consequently, addressing social isolation is essential when targeting older people living in deprived areas. It is also possible that, despite the benefits of social stimulation, home-based health promotion might be an option (at least initially) to be able to reach older people in deprived areas. Further, poor literacy was reported by both some older people in deprived areas and professionals with experience working with them. Low levels of literacy are often associated with older people from BME groups [32], suggesting literacy problems could be given more attention among older people in deprived areas too.

Concerns about the health and wellbeing of older people living in deprived areas have not been reflected in existing policy in England [33]. Except for efforts on national level such as addressing fuel poverty and increasing uptake of the influenza vaccine, there are few examples of practical health promotion interventions specifically targeting older people living in deprived areas [32]. A recent international study concluded that health promotion interventions for older people in European countries are rarely implemented at a national level and tend to lack sustainable funding [35]. In England, some urban and rural areas have addressed costs and availability of transport to enable older people to take up services [32], also reported by professionals in the current study as an enabler for engaging older people from deprived areas in health promotion. Older people in deprived areas are among the most vulnerable populations in England [33], and health promotion interventions targeting this group need to be prioritised to address health inequalities in later life [3].

### Strengths and limitations

Strengths of this study include that we successfully recruited and captured the views of 19 older men and women from three different ‘hard to reach’ groups, 12 of whom had not had blood pressure checks or influenza vaccination in the last two years. We also consulted 31 professionals with work experience from these groups across various sectors. Limitations of the study include that the data of participating older people ‘hard to reach’ obtained through interviews and focus group were richer than the survey data from the professionals. Professionals who chose to complete the survey were self-selected and we may not have captured a wide range of professionals. Those who participated were motivated to complete the survey and may have comprehensive knowledge of their field, possibly explaining their high level of consensus. Similarly for the interviews with older people, those participating may be more motivated and engaged than those who did not take part, and this might particularly apply in the group of the oldest old where uptake of invitations to take part from primary care were low, and three of the five interviewed were attending a day centre. Furthermore only one of five of the oldest old was a man, and the views may not reflect those of older men in this group. Also, although participating older people from BME groups included Black African Caribbean, Asians and Europeans, most of the Europeans came from Ireland and all Asians were from Bangladesh suggesting some BME groups were under-represented and their views may be different. Four of the older people from BME groups further formed a focus group rather than participated in individual interviews. Sharing their views in a group of people they know may have restricted them speaking openly. However, it may also have had the opposite impact, making them feel more confident [36]. In our study the majority of participating older people belonged to more than one ‘hard to reach’ group, making it difficult to allocate findings to one specific ‘hard to reach’ group. Furthermore, participating older people living in deprived areas were often engaged in activities themselves but reported that their neighbours/peers rarely leave their homes. Thus, despite efforts to sample widely, we may not have reached the most vulnerable older people in deprived areas. Another limitation is that participants were not invited to provide feedback on the findings. However the interpretations of the findings resonated with lay members who were part of the research team. The professionals recruited completed an anonymous survey with no opportunities for follow-up, restricting the data. However their responses had high consensus and provided further insights to the findings from the interviews and focus group with the older people deemed ‘hard to reach’.

## Conclusions

Based on the views of ‘hard to reach’ older people (oldest old, older people from BME groups and older people living in deprived areas) and the views of cross-sector professionals with work experience of these groups, there are some shared characteristics and circumstances across the three ‘hard to reach’ groups but also some clear differences. This suggests that a carefully outlined strategy should be considered to successfully reach those targeted. Older people from BME groups and older people living in deprived areas reported being reluctant to access health promotion. Future interventions are needed to investigate whether empowering older people from BME groups and those living in deprived areas has a positive impact on their attitude towards health promotion. These two ‘hard to reach’ groups further reported some poor literacy and language problems (BME only) which suggests that any written information should also be provided verbally, using an interpreter if needed. Additionally, offering free activities and continuity in activities may increase engagement in health promotion activities among older people living in deprived areas. Further research is also needed to examine whether home-based health interventions, known for facilitating health interventions among the oldest old, also could improve the health and wellbeing of older people living in deprived areas.

## Additional files

Additional file 1: Interview topic guide. Questions asked to participating older people. (DOCX 93 kb)

Additional file 2: Expert consultation questionnaire. Questions asked to participating professionals. (DOCX 54 kb)

## Abbreviations

BME: Black and Minority Ethnic
GP: General Practitioner
NHS: National Health Service
US: United States of America
