# Supplementary material for: Engaging ‘hard to reach’ groups in health promotion: the views of older people and professionals from a qualitative study in England
Source: BMC Public Health. 2019 May 23;19:629. doi: 10.1186/s12889-019-6911-1 (PMC6533740; doi:10.1186/s12889-019-6911-1)
Supplement: Supplementary file 1 — Interview topic guide. Questions asked to participating older people. (DOCX 93 kb) [file 12889_2019_6911_MOESM1_ESM.docx]

**Healthy Ageing, Research and Participation**

**Topic guide for focus groups/interviews with older people**

Checklist:

- Read and understood information sheet?
- Opportunity to ask questions
- Confidentiality and exceptions to this
- Information about types of questions will be asking, no right or wrong answers
- Consent form

1. Thoughts on engaging in things that help people stay as healthy and feel as well as possible as they become older.

Probe:

- What does ‘being healthy’ mean to you?
- Can you tell me a little bit about your current health and well-being *(feeling as well as possible, being able to do things that are important to them, staying independent)*
  - What is a ‘good day’ for you?
  - What kinds of things are important for you to be able to keep doing?
  - What helps you to maintain this?
  - Is anything holding you back from feeling well? Why?
- Identify what is most important in feeling as well as possible. What makes a ‘good day’?
  - What helps? – why?
  - What gets in the way of this?
  - Can you give an example of something that has made a difference to your health *(advice from professionals, support from family/friends, health campaigns)*
    - Why did this help / not help?
    - Probe on other services they do visit *(e.g. dentist, opticians)* – why do you attend them?
- Experiences / examples of any advice given that has / has not been taken on
  - Decision making involved in this.
  - Probe more on why don’t see their GP. What stops them? What would help?
  - How do they seek help in general? Where? How often? At which point?
- How they compare to other people they know with the same background / age/health circumstances as them.
  - If different – why?
- What things have you done throughout your life to stay well? *(probe on what has helped / hindered throughout life course, and whether still doing these things?)*
- Follow-up any mention of friends/family/social circle: does this influence their health/health decisions? Where do you get your information/support from?
  - - - SUMMARISE SECTION

1. Support needed to be healthy as possible as you get older.

Probe:

*(Gauge following responses to earlier questions)*

- Have you had any support to maintain OR improve your current health/happiness as it is now?
  - Experience of this and could anything be done better? If not, what do you think would be helpful?
  - Looking to the future – what issues do you think you might have in 5 years time? What might help?
- What kind of support would you find helpful? *(to maintain/improve health)*
  - Setting? *(GP surgery, home, somewhere else)*
  - Details of potential intervention or support?
  - Who might be the most influential person in making a recommendation to improve health – reduce risks of disability or illness (*e.g. GP/nurse, religious/community leaders/venues, family members, peers, friends, neighbours, etc.),*
  - Other sources? *(e.g.TV adverts, other media, verbal vs written vs email or text)*
  - Whose opinion matters to you / whose advice would you seek?
  - What would encourage you to take part / engage with this support? *(e.g. time of day, transport, single sex, club type atmosphere, etc)*
  - What might discourage you from taking part? *(e.g. transport, getting back in the dark, toilet accessibility etc.)*
    - - SUMMARISE SECTION

1. Thoughts on taking part in research:

Probe:

- - What is your understanding of ‘research’? What does the word ‘research’ mean to you?
  - Thoughts about *research (value, effort, time, understandability, feeling of being a ‘guinea pig’, worry about what might be found)*
    - What made you take part this time?
    - What do you think is key to taking *part (e.g. home-based, voucher)*?
- Any experience of being involved in research, being asked and agreed/not agreed
  - Reasons and thoughts about this
  - Anything that worries you about taking part in research? – why?
  - Thoughts about research specifically on health promotion for older people *(e.g. health beliefs, fatalism etc)*
    - How important do you think it is to do this kind of research on the health of older people? – why?
  - If reluctant to take part – why?
  - If keen to take part, what is/would be best approach?
    - What do you think would help more people take part in research like this? – why?

– SUMMARISE SECTION

- **Demographics form (next page)**

Participant ID:

**About You**

1. What is your date of birth?

…………………………………………………………………...

1. Where were you born?

1. United Kingdom
2. In another country (please specify)

……………………………………………………………

1. What is your ethnic group?

White

1. English/Welsh/Scottish/Northern Irish/British
2. Irish
3. Gypsy or Irish Traveller
4. Any other white background: …………………………

Mixed/multiple ethnic groups

1. White and black Caribbean
2. White and black African
3. White and Asian
4. Any other mixed/multiple ethnic backgrounds:…………………………………………

Asian/Asian British

1. Indian
2. Pakistani
3. Bangladeshi
4. Chinese
5. Any other Asian background:………………………….

Black/African/Black Caribbean/Black British

1. African
2. Caribbean
3. Any other black/African/Caribbean background:……………………………………………..

Other ethnic group

1. Arab
2. Any other ethnic group:………………………………..
3. What are your current living arrangements?
4. I live alone
5. I live with my spouse/partner
6. I live with another family members/other family members
7. I live with a friend/someone else
8. Other (please describe) ……………………………….
9. What is your current marital/civil partnership status?
10. Single, never married/civil partnership
11. Co-habiting with partner
12. Married or in a civil partnership
13. Separated, but still legally married/in a civil partnership
14. Divorced
15. Widowed
16. Which of the following best describes your current housing?
17. Owner-occupied
18. Council rented
19. Housing Association rented
20. Social housing rented
21. Private rented
22. Sheltered housing (specify whether council/housing association/social housing/private)
23. Other (please specify) …………………………………
24. At what age did you complete your education in school or college?
25. Before the age of 15 years
26. At the age of 15 or 16 years
27. Between the ages of 17 and 20 years
28. After the age of 21 years
29. Are you currently employed?
30. Yes, full-time
31. Yes, part-time
32. No
33. What type of pension do you receive (please tick all that apply)?
34. State pension
35. Employer pension
36. Private pension
37. Not applicable
38. Do you receive any of the following benefits (please tick all that apply)?
39. Pension credit
40. Disability Living Allowance or Attendance Allowance
41. Housing benefit
42. Council Tax benefit
43. Any other benefits (please specify)………………….
