# Supplementary material for: Engaging ‘hard to reach’ groups in health promotion: the views of older people and professionals from a qualitative study in England
Source: BMC Public Health. 2019 May 23;19:629. doi: 10.1186/s12889-019-6911-1 (PMC6533740; doi:10.1186/s12889-019-6911-1)
Supplement: Supplementary file 2 — Expert consultation questionnaire. Questions asked to participating professionals. (DOCX 54 kb) [file 12889_2019_6911_MOESM2_ESM.docx]

**Healthy Ageing, Research and Participation**

**Expert Consultation**

**We are interested in gathering your views and experiences of engaging older people who may be less likely to take part in health promotion and research on ageing well.**

*Please complete the questions below:*

1. Which of the following groups do you have experience of engaging with, either for health promotion or research on ‘ageing well’? (please tick all those that apply)

Please indicate whether you work primarily in healthy ageing research or health promotion.

- 1. Older people aged 85 years and over

Healthy ageing research

Health promotion

- 1. Older people (65+ years) living in more deprived neighbourhoods

Healthy ageing research

Health promotion

- 1. Older people (65+ years) from black and minority ethnic (BME) groups

Healthy ageing research

Health promotion

- 1. Other groups of older people who might be less likely to take part in healthy ageing activities (please give details below)

…………………………………………………..…………………………………………………......................................................................................................................

Healthy ageing research

Health promotion

[new section]

**Older people aged 85 years and over**

*If you do not have experience of engaging with this group, please go to question 5.*

1. In your experience, what **helps or** **facilitates** engagement with older people aged 85 years and over? Please give examples.
2. In your experience, what are the **barriers or difficulties** of engaging with this group? Please give examples.
3. How do you think these **barriers or difficulties** could be **overcome**?

[new section]

**Older people (65+ years) living in deprived neighbourhoods**

*If you do not have experience of engaging with this group, please go to question 8.*

1. In your experience, what **helps or** **facilitates** engagement with older people (65+) living in deprived neighbourhoods?
2. In your experience, what are the **barriers or difficulties** of engaging with this group? Please give examples.
3. How do you think these **barriers or difficulties** could be **overcome**?

[new section]

**Older people (65+ years) from black and minority ethnic (BME) groups**

*If you do not have experience of engaging with this group, please go to question 11.*

If you have experience of more than one BME group, please highlight any differences between the groups (if appropriate):

1. In your experience, what **helps or facilitates** engagement with older people from BME groups? Please give examples.
2. In your experience, what are the **barriers or difficulties** of engaging with these groups? Please give examples.
3. How do you think these **barriers or difficulties** could be **overcome**?

[new section]

**About you**

1. What sector do you (primarily) work in?

NHS

Local authority

Local government

Social care

Private

Academic

Voluntary/third sector

Other, please specify

1. What is your job title?

Director

Manager

Project officer/coordinator

Researcher/lecturer

Commissioner

Practitioner

Other, please specify

**Thank you very much for taking your time to answer the questions.**
